# Hepcidin is potential regulator for renin activity

**DOI:** 10.1371/journal.pone.0267343

**Published:** 2022-04-20

**Authors:** Jaakko Piesanen, Jarkko Valjakka, Sanna Niemelä, Marjut Borgenström, Seppo Nikkari, Vesa Hytönen, Juha Määttä, Tarja Kunnas

**Affiliations:** 1 Facult of Medicine and Health Technology, Tampere University, Tampere, Finland; 2 Patendo Oy, Parainen, Finland; Universitat zu Lubeck, GERMANY

## Abstract

An association between genetic variants in the genes *HFE*, *HJV*, *BMP4* and arterial hypertension has been shown earlier. Proteins encoded by these genes participate in the signalling routes leading eventually to the production of the peptide hormone hepcidin. Mutations in these genes have been associated with the abnormal production of hepcidin in the body. This finding led to studies exploring the possible role of hepcidin in regulating the activity of blood pressure related renin-angiotensin system enzymes. We used molecular modelling to find out if it is possible for hepcidin to bind to the active site of the renin-angiotensin system enzymes, especially renin. Fluorometric assays were used to evaluate the inhibitory effect of hepcidin on renin as well as angiotensin converting enzymes 1 and 2. Finally, bio-layer interferometry technique was used to study hepcidin binding to renin. The molecular modelling showed that hepcidin seems to have similar binding properties to the renin active site as angiotensinogen does. Based on fluorometric enzyme activity assay, hepcidin has an inhibitory effect on renin *in vitro*, too. However, angiotensin converting enzymes 1 and 2 were not inhibited remarkably by hepcidin-25. In bio-layer interferometry analysis hepcidin-renin binding was concentration dependent. Our results suggest that hepcidin could act as an inhibitor to the renin. Nowadays, there is no known biological inhibitor for renin *in vivo* and our finding may thus have important clinical implications.

## Introduction

Hepcidin is a peptide hormone known to be the main regulator of systemic iron homeostasis, but it has antimicrobial activity in the body, too. Hepcidin production is stimulated by inflammation and elevated serum iron concentrations, whereas erythropoiesis and decreased amount of iron in the circulation suppress the expression of hepcidin. It is mainly produced in liver but some expression has also been detected in heart, kidney, skeletal muscle, adipose tissue, and brain suggesting a role independent of the systemic hepcidin regulation [1–3].

The regulation of hepcidin transcription is complex and at least three pathways are leading to transcription initiation. Hemochromatosis protein, hemojuvelin, transferrin receptor 2 and bone morphogenetic protein receptors coordinate signalling through SMAD-pathway leading finally into the nuclear translocation of SMAD proteins and activation of hepcidin transcription [1, 3]. Transcription is also upregulated during inflammation and infection by IL-6 through the STAT3 pathways [1] and suppressed during hypoxia via the hypoxia-inducible factor (HIF) pathway. HIF-1 has been suggested to be a direct suppressor of hepcidin gene *HAMP* [4] but subsequent studies have revealed *HAMP* suppression to occur via HIF-2-dependent induction of erythropoietin production [5, 6]. After maturation, active 25-amino acid hepcidin is secreted into the circulation [7].

Many human diseases are associated with alterations in hepcidin concentrations of which probably the most significant are anaemia of chronic disease and hereditary haemochromatosis [1]. In addition, dysregulated iron metabolism has been linked to cardiovascular diseases as well. Hepcidin’s role in the pathogenesis of atherosclerosis has recently been investigated with conflicting results [8]. In the LURIC study including almost 2000 patients, low hepcidin levels were found to associate with higher cardiovascular and all-cause mortality [9]. Valenti et al. also reported that low circulating hepcidin concentrations associated with high aortic stiffness [10]. However, results from Nijmegen Biomedical Study indicate possible causative role of increased hepcidin levels to atherosclerosis [11, 12]. There is only one study so far where circulating hepcidin levels were found to associate systolic blood pressure especially in men [13].

Previously, we have shown an association between genetic variants of *HFE*, *HJV*, *BMP4* and arterial hypertension [14–16]. Also, Selvaraj et al. [17] and Ellervik et al. [18] have found similar effect in hypertension prevalence between different *HFE* genotypes carriers. As the proteins, HFE, HJV and BMP4 participate in the signalling routes leading eventually to the production of the hepcidin, we decided to examine a possible relationship between hepcidin and blood pressure regulation system. Experiments where angiotensin II infusion affected hepcidin mRNA expression in mice [19, 20] encouraged us to focus on the renin-angiotensin system (RAS) and hepcidin interaction studies.

The RAS has a central role in the physiological blood pressure regulation system as well as in the pathophysiology of hypertension. In this study, we wanted to find out whether it is possible for hepcidin to interact and inhibit the enzymatic activity of the RAS enzymes, especially renin.

## Materials and methods

### Computational molecular modelling

Computational, structure-based methods CABS-dock [21], ZDOCK [22] and Yasara minimization server [23] were used to determine how the hepcidin hormone (hepcidin-25) binds to the renin as well as angiotensin converting enzymes 1 (ACE1) and 2 (ACE2). Methods are based on the normal enzyme-substrate/inhibitor interactions. The modelled protein structures were crystal structures of the complex of human angiotensinogen and renin [24] (PDB ID: 6I3F), and hepcidin-25 [25] (PDB ID: 4QAE) from Protein Data Bank [26]. Hepcidin-25 used in the modelling contained four sulphur bridges and thus had its intrinsically functional intact molecular structure. CABS-dock does not feature peptides’ disulphide bonds and therefore, we used ZDOCK web server for peptide-protein docking to the results. Selected docking models were refined with molecular dynamics simulation, using NAMD on Sisu supercomputer at CSC–IT Center for Science Ltd. SWISS-MODEL [27] and UCSF ChimeraX [28] programs were used to create the structure models for visualization and analysing.

### Measurement of renin activity

Activity measurements for all three enzymes (renin, ACE1 and ACE2) were carried out using commercial inhibition kits following manufacturer’s instructions. Screening kits were purchased from Biovision (Biovision, Inc. San Francisco, CA). Candidate inhibitors used were human hepcidin-25/LEAP-1 (disulfide bonds between Cys7–Cys23, Cys10–Cys13, Cys11–Cys19, and Cys14–Cys22, purity assessed by HPLC ≥ 98.0%) purchased from Peptides International (Peptides International Inc, Louisville, KY) as well as N-terminal part of hepcidin, Asp-Thr-His-Phe-Pro (hepcidin-5) synthetized by Proteogenix (Schiltigheim, France).

All kits included the enzyme, substrate, and enzyme inhibitor as negative control. The fluorophore labelled substate was cleaved by the enzyme. The used fluorophores were a synthetic o-aminobenzoyl peptide for ACE1, 7-Methoxycoumarin-4-acetic acid (MCA) for ACE2 and 5-((2-Aminoethyl)amino)naphthalene-1-sulfonic acid (EDANS) for renin. DABCYL-EDANS is well-known FRET pair where fluorescence is quenched when peptide is uncleaved. Fluorescence signal is increased after peptide is cleaved and EDANS molecule is free in the solution. The fluorescence intensity was recorded and calculated as follows. According to renin inhibitor screening kit instructions hepcidin-25 and hepcidin-5 were first pipetted to a black ForteBio’s 96-well plate. Renin enzyme solution was added and mixed. Both candidate inhibitors were measured with final sample concentrations of 10 nM, 100 nM and 500 nM. The plate was incubated for 5 min at 37°C. Finally, substrate solution was added into each well. Fluorescence measurement was performed using Perkin Elmer UV/VIS Envision multimode plate reader. Excitation and emission wavelengths were 328 and 552 nm, respectively. Fluorescence was recorded every 60 s for 60 min at 37°C. Inhibitory activity of renin was calculated as follows:

$$Renin\;inhibitory\;activity\;(\%)=1-\frac{\mathrm{Activity}\;\mathrm{with}\;\mathrm{sample}}{\mathrm{Activity}\;\mathrm{without}\;\mathrm{sample}}x\;100$$

where “Activity with sample” is the slope calculated from the linear range of the fluorescence plot at the presence of candidate inhibitor. Activity without the sample represents the slope without any sample in the well.

### Measurement of angiotensin-converting enzyme 1 activity

Briefly, ACE1 enzyme solution was added to a 96-well plate and each diluted candidate enzyme inhibitors were added. The plate was incubated for 15 min at 37°C before adding substrate solution. Excitation and emission wavelengths were 330 nm and 430 nm, respectively. Fluorescence was recorded every 60s for 100 min at 37°C. Calculation of inhibitory activity for each sample followed the equation of the renin kit.

### Measurement of angiotensin-converting enzyme 2 activity

ACE2 enzyme solution was added to a 96-well plate, each diluted candidate inhibitors were added, and the plate was incubated for 15 min at room temperature before adding substrate solution. Fluorescence was recorded every 60 s for 60 min at room temperature with the excitation and emission wavelengths of 320 nm and 420 nm. Inhibitory activity calculation followed the equation above.

### Renin production and purification for bio-layer interferometry analysis

Human prorenin coding gene was ordered from GeneArt in pcDNA3.1(+) as fusion protein with a C-terminal His-tag. The pcDNA3.1 vector confers ampicillin resistance. Plasmid was dissolved in 50 μl of TE-EF and the amount of 100 ng was transferred into Escherichia coli TOP10 cells (Invitrogen, UK) with the heat shock method (30 s). In the next day colonies were picked for midiprep isolation. A single colony was picked and cultured in 5 ml media supplemented with 100 μg/ml ampicillin and 5 mM glucose for 7 hours after which it is moved to 200 ml LB media, which included ampicillin and glucose, and grown overnight at 37°C 220 rpm. The plasmid DNA was isolated using Nucleobond Xtra Midi Plus EF Kit (Macherey-Nagel, #7404220). Plasmid DNA produced was endotoxin-free. Human prorenin was then produced in ExpiCHO cells.

Cell pellet was re-suspended in 20 ml buffer (50 mM Hepes, 200 mM NaCl, 10% Glycerol, 1 mM TCEP, pH 7.5) and protease inhibitor tablet was added. Cell lysis was made with sonicator (1 min, 50%, 2 sec on/5 sec off cycle). Cell debris was separated (appeared black after sonication) by centrifugation and the supernatant was purified with metal affinity chromatography. The supernatant (30 ml) was bound to 1 ml of Co-NTA agarose in a batch mode for 1.5 h at 4°C. After incubation, the resin has lost its red colour and turned opaque white. The bound protein was washed with 20 CV (40 ml) of buffer (50 mM Hepes, 200 mM NaCl, 10% Glycerol, 1 mM TCEP, pH 7.5) followed by 4 CV (8 ml) steps of elution with increasing concentration of imidazole from 5, 10, 20, 30, 40, 50, 100, 200, and 300 mM. The resin remained white throughout the entire protocol, and it was washed with 1 M NaOH and recharged with fresh Co^2+^ after use which turned the colour back to original red. Renin was achieved by proteolytic cleavage of a 43 amino acid N-terminal prosegment using limited enzymatic digestion by immobilized trypsin.

### Biosensor analysis of hepcidin—Renin binding

Biotinylation of renin was done using NHS-PEG4-Biotin (ThermoFisher Scientific) at 1:1 molar ratio. More precisely fresh 2 mM NHS-PEG4-Biotin was added to the protein solution and mixture was incubated on ice for 2 hours. Thereafter, free biotin was removed with Zeba desalt spin columns (ThermoFisher Scientific). Concentration of biotinylated protein was measured using NanoDrop™ One Uv-Vis spectrophotometer (ThermoFisher Scientific). Biologically active hepcidin-25 binding to the produced human renin was confirmed using bio-layer interferometry technique. Hepcidin-20 (purchased from Pepnet.com/Detail/1838/Hepcidin-20-(Human) was used as a control peptide. Analysis was carried out on a ForteBio Octet RED384 instrument (Pall Life Sciences) using nickel- nitrilotriacetic acid sensors. Samples and buffers were prepared into 96- or 384-well plates. Sensors were prewetted with the buffer (PBS pH 7.2 and 150 mM NaCl) to get baseline measurements prior to protein immobilization. An operating temperature of 27°C and a stirring speed of 1,000 rpm were used throughout the experiment. Sensors were chemically activated by immersion in 0.05 M 1-ethyl-3-(3-dimethylaminopropyl)carbodiimide and 0.1 M N-hydroxysuccinimide in water for 100 s. Both commercial renin (BioVision) and in house made renin was biotinylated and used for biolayer interferometry experiments. In preliminary measurements, biotinylated commercial renin and biotinylated in-house made renin was immobilized on the streptavidin sensors using 2.5, 5, and 10 μg/ml and 20 μg/ml, respectively. Purpose of different concentrations of was to find best possible loading concentration for kinetic measurements. Serially diluted hepcidin-25 were applied on the renin-coated sensors in concentrations of 12.5, 25, 50 and 100 μM to obtain the relative interaction of hepcidin-25 to renin.

### Statistical analysis

Mean enzyme inhibitory activities with the standard error of means were calculated at different concentrations. Data analysis was performed using Microsoft Office 365 ProPlus Excel version 1902 for Windows. The hepcidin–renin binding data was treated as follows: Background was subtracted, and the data was normalised to allow comparison between experiments. The normalised data was analysed with linear regression analysis in GraphPad Prism 5.02 (GraphPad Software, La Jolla, CA) assuming one-site specific binding.

### Ethics statement

Ethical approval was not sought because the study was carried out using computational and laboratory technical methods without human or animal tissues. Informed consent was not needed since no patient information was included in the study.

## Results

**Fig 1:**
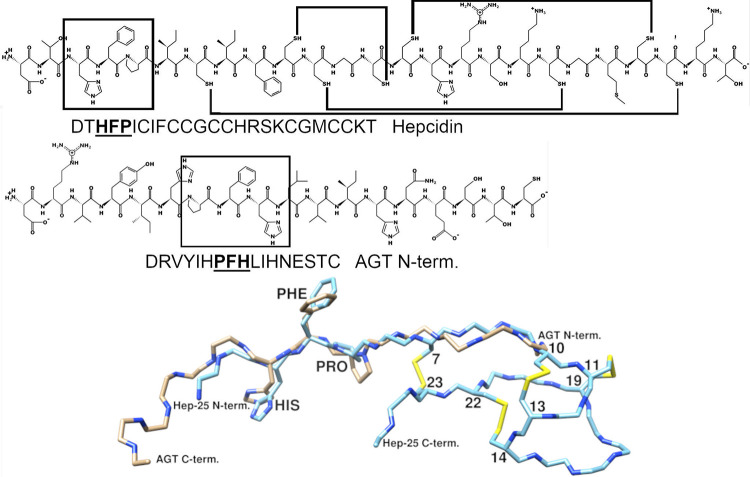
Similarity of amino acid sequences and structural formulas of hepcidin hormone and N-terminal part of the human angiotensinogen. A similar binding motif and its amino acids histidine, phenylalanine and proline are framed and written in bold. The 3-dimensional structure of Hep-25 (chain from PDB notation 4qae), and the N-terminal structure of the human angiotensinogen (chain from PDB notation 6i3f) are coloured with cyan and brown respectively. In addition, the four disulfide bonds of hepcidin are in yellow and their cysteine amino acids are numbered.

**Fig 2:**
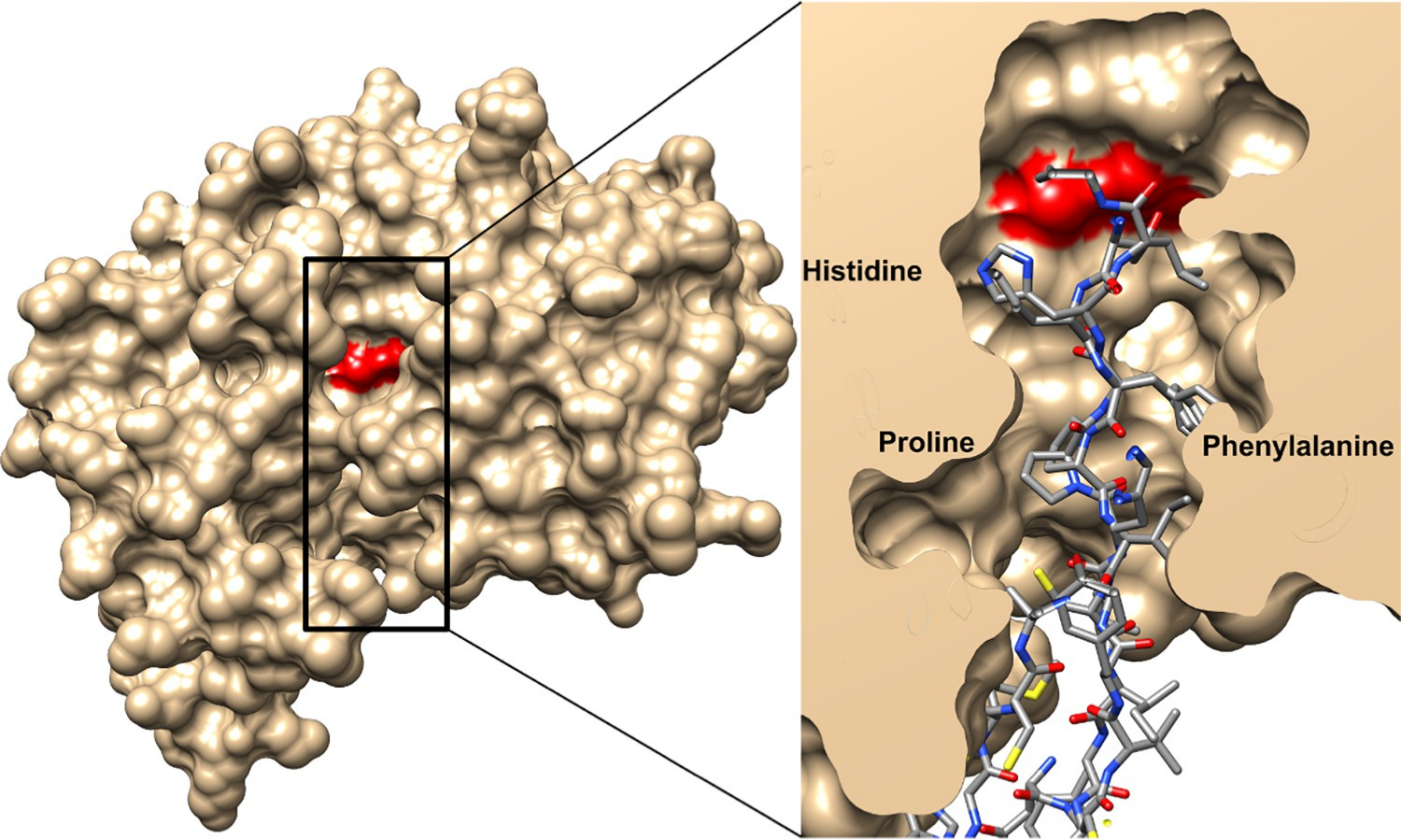
The molecular surface representation of human renin and its activity site. Superposition of human hepcidin and N-terminal angiotensinogen peptides and surface representations indicate common structural position of histidine, phenylalanine, and proline motifs in renin active site. This representation shows moreover, how hepcidin binding near the catalytic aspartates (in red) can cause renin inhibition.

In molecular modelling we found that N-terminal parts of hepcidin-25 and angiotensinogen have similar binding motif Pro-Phe-His. This motif is located near to the peptide bond which is the target for renin. Therefore, it likely nestles in the same way on the active site of the renin although the motif occurs reversed in the polypeptide chain of hepcidin-25. We used structure-based molecular docking methods to evaluate whether it is possible for different hepcidin peptides to enter the active site of renin. Structures of hepcidin-25 and angiotensinogen are shown in Fig 1. For peptides to undergo binding, they must collide and take orientations that enable them to bind together by noncovalent interactions. Hepcidin-25 has two regions which are involved in the binding to renin. The first is a flexible N-terminal structure (amino acids 1–6), which, when bound into the active site of renin, prevents renin´s normal proteolytic activity. This critical part is assisted into the active site by another compact fold (amino acids 7–25) representing the second region important for binding to renin. Together these two parts create suitable contacts for interaction with renin. The hepcidin inhibition may be competing because its important amino acid motif (His-Phe-Pro) can settle in the same positions as the corresponding amino acids of the endogenous angiotensinogen substrate of renin. Notably, respective orientation of these competing peptides is opposite as illustrated in Fig 1. Based on the modelling, we propose that the N-terminal part of hepcidin-25 could bind to the active site of renin and therefore have an inhibitory effect on it. Further, superposition of hepcidin-25 and angiotensinogen main chains atoms and binding surface representations indicate common structural positions for the His-Phe-Pro -motifs in the active site, in Fig 2.

Further, the inhibitory effects of hepcidin were confirmed *in vitro*. Commercial screening kits for RAS enzymes were used to evaluate possible renin, ACE1 and ACE2 enzyme inhibition by hepcidin. In addition to the hepcidin-25, we used hepcidin-5 to study its potential in the inhibition of the RAS enzymes. Although hepcidin-5 has not been detected *in vivo*, we tested it *in vitro*, because it contains an amino acid triad important for binding to the active site of renin.

**Fig 3:**
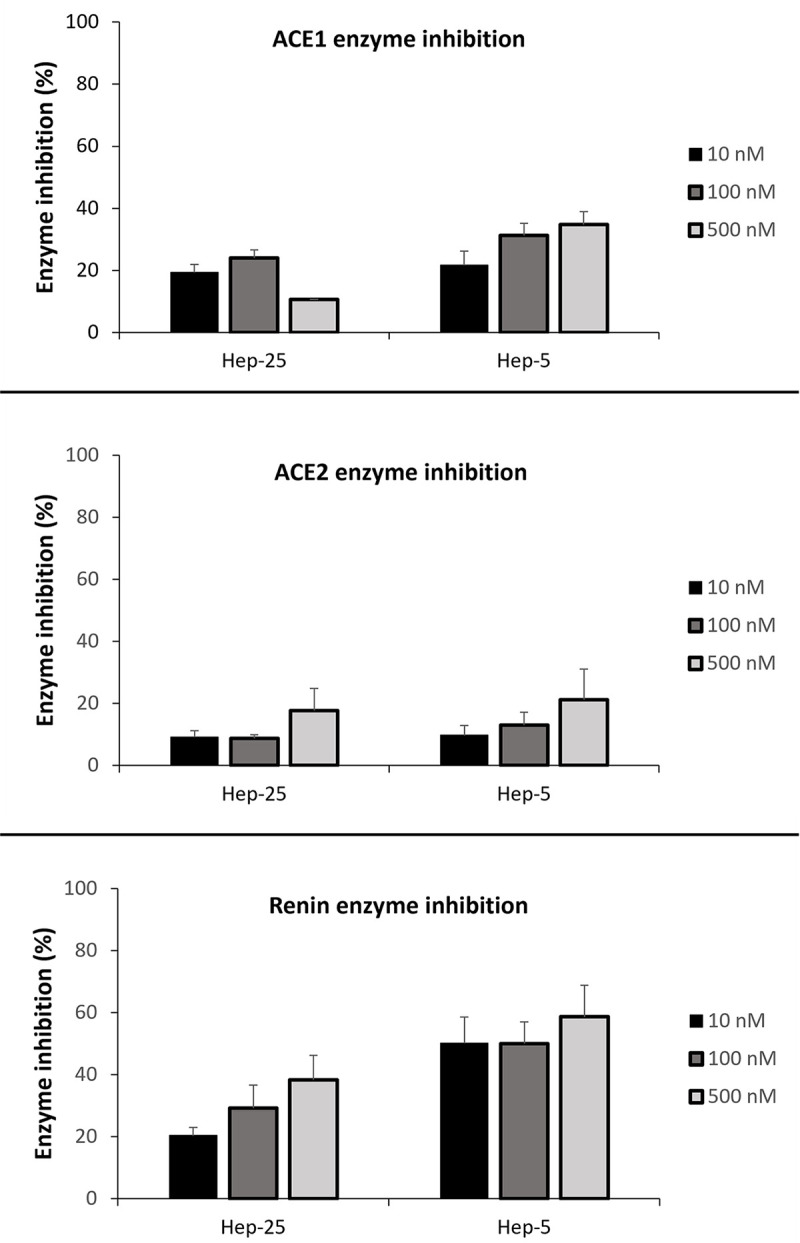
Inhibitory effects of hepcidin-25 and hepcidin-5 on the ACE1, ACE2 and renin with the standard errors of mean (SEM). Fluorometric enzyme inhibition analysis was measured with peptide concentrations 10, 100 and 500 nM. Hepcidin peptides had the highest inhibitory effect on renin. Number of experiments for concentrations 10 nM, 100 nM and 500 nM, respectively. ACE1 and hepcidin-25: 5-6-1. ACE1 and hepcidin-5: 4-6-6. ACE2 and hepcidin-25: 3-6-4. ACE2 and hepcidin-5: 3-6-3. Renin and hepcidin-25: 2-3-3. Renin and hepcidin-5: 2-3-3.

Mean inhibitory effects of hepcidin-25 on renin activities were 20.4%, 29.2% and 38.3% with concentrations of 10 nM, 100 nM and 500 nM, respectively. For hepcidin-5, the corresponding inhibitory effects were 50.1%, 50.0%, and 58.6%, Fig 3. In the case of ACE1, hepcidin-25 had a low inhibitory activity, whereas hepcidin-5 had its strongest inhibitory activity (34.8%) at the concentration of 500 nM. No significant inhibition was found when the peptides were incubated with ACE2, as shown in Fig 3.

**Fig 4:**
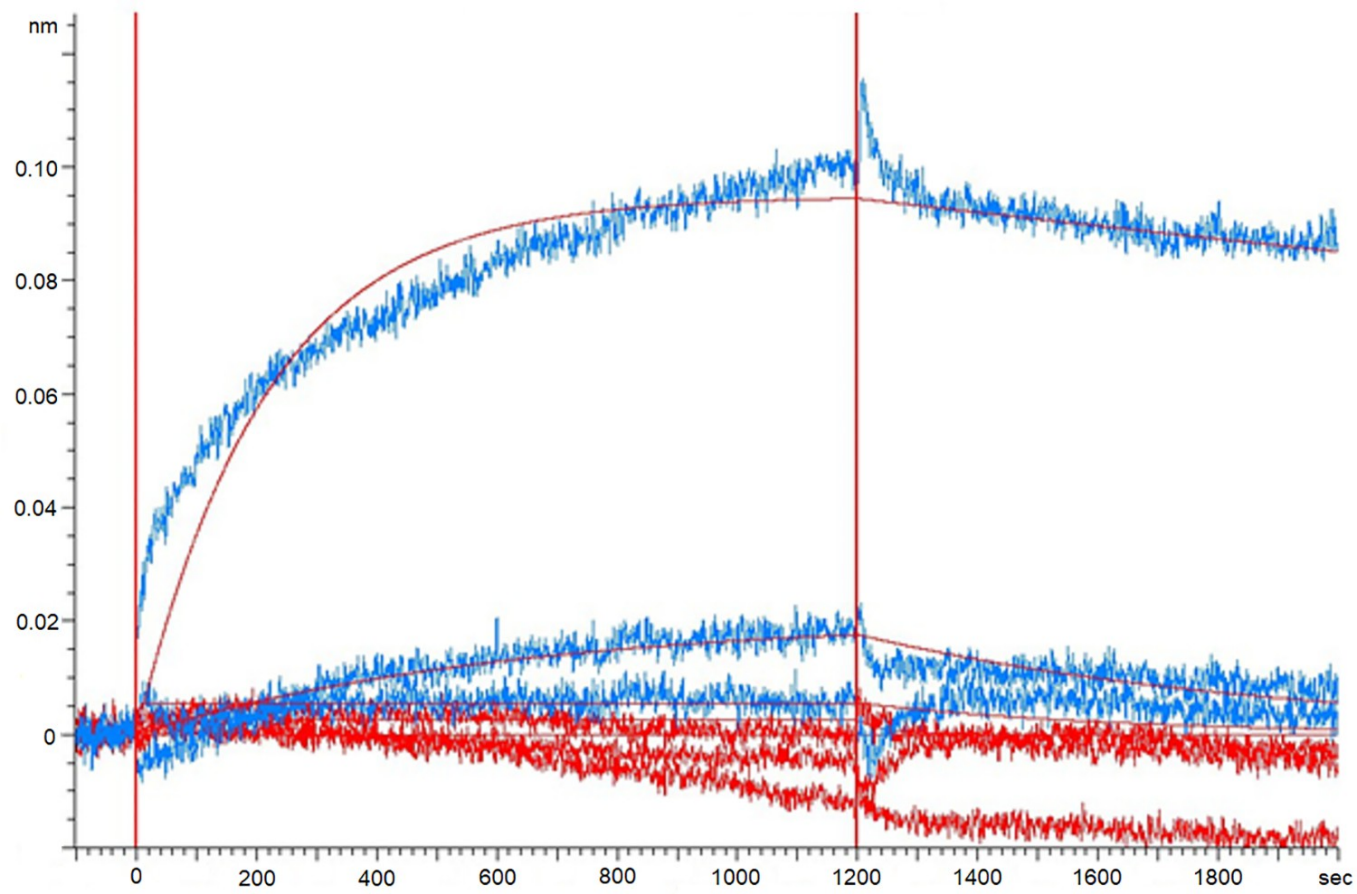
Kinetic measurements of renin and hepcidin-20 and hepcidin-25. Binding properties of different forms of hepcidin was measured with biolayer interferometry using biotinylated renin as ligand with streptavidin sensors and hepcidin-20 and hepcidin-25 as analyte. Binding was measured with different concentrations of analyte (100, 50 and 12.5 μM) and binding curves of hepcidin-25 and hepcidin-20 are shown with blue and red, respectively. Baseline (-100–0 s), association (0–1200 s) and dissociation (1200–2000 s) are shown.

Biologically active hepcidin-25 binding to the produced human renin was confirmed using bio-layer interferometry technique. The association and dissociation curves indicated that hepcidin-25 binding to renin is concentration dependent. In addition, the measurements showed that hepcidin-20 cannot bind to renin because of the absence of N-terminal hepcidin-5 in the structure. Thus, the motif (His-Phe-Pro) is important to hepcidin binding, Fig 4.

## Discussion

We have discovered that biologically active hepcidin can bind to renin enzyme and inhibit its activity *in vitro*. Using molecular modelling we observed that hepcidin binds to the active site of renin near the catalytic amino acids Asp38 and Asp226. When the amino acid sequences of hepcidin and natural renin substrate angiotensinogen were compared it was found that these peptides have similar binding motifs for renin. After this observation, we made renin inhibition measurements using commercial renin inhibitor screening kit. Since the inhibition was obtained with the kit, we wanted to make sure that engagement is not an artefact. Therefore, we produced a larger amount of renin for bio-layer interferometry analysis to confirm that hepcidin binding to renin is concentration dependent. Nowadays, there is no known biological inhibitor for renin *in vivo*, and our findings may thus have important clinical implications.

In addition to the hepcidin-25, we used hepcidin-5 to study its potential in the inhibition of the RAS enzymes. Although hepcidin-5 has not been detected *in vivo*, we tested it *in vitro* because it contains an amino acid triad important for binding to the active site of renin. The inhibition provided by hepcidin-5 confirmed the results. The hepcidin-5 had a stronger effect on renin than hepcidin-25, which can be explained by the differences between their molecular sizes and the shapes of hepcidin-25 and the hepcidin-5. The hepcidin-25 is five times larger in amino acid volume than the hepcidin-5. Moreover, hepcidin-25 contains four covalent sulphur bonds making it more globular, while the hepcidin-5 occurs as a linear peptide. Campostrini and co-workers have studied serum levels of hepcidin-25 and its truncated isoforms [29]. They found that in addition of hepcidin-25, also detectable amounts of hepcidin-20 were found in human sera. In addition, these truncated forms have been found in plasma samples obtained from patients suffering from diseases with significantly increased hepcidin concentrations, such as sepsis, kidney failure and the acute phase of myocardial infarction [29–31] but their exact biological meaning is unknown. Also, the enzymes responsible for the creation of these isoforms are not known. However according to our bio-layer analysis hepcidin-20 cannot bind to renin suggesting the importance of the hepcidin-25’s N-terminal part in binding to renin.

Renin is the specific protease of the RAS system that cleaves an inactive peptide angiotensin I from the plasma protein angiotensinogen. So far, angiotensinogen has been the only known endogenous substrate for this blood pressure controlling enzyme. Highly potent vasoconstrictor peptide angiotensin II is further cleaved from the angiotensin I. Cleavage occurs especially in the endothelial cells of the lungs by the non-specific protease ACE1. If hepcidin turns out to be the biological inhibitor of renin preventing angiotensinogen’s binding *in vivo*, it will bring new information for the blood pressure control system.

Although our main interest was aimed at renin, we wanted to study the inhibitory properties of hepcidin on ACE1 too. The N-terminal part of hepcidin is close to the angiotensin I in terms of its binding properties. According to our models it is possible that hepcidin-5 may bind weakly to the active site of ACE1 with two orientations. In both directions, N-terminus glutamate amino acid is in near contact with catalytic cofactor zinc in the ACE1. This may explain *in vitro* results of ACE1 inhibition by hepcidin. ACE2 was included as a reference enzyme since our molecular modelling showed that no specific binding would probably occur. Inhibitory measurements confirmed this, since only weak inhibition was observed.

Iron related metabolism and blood pressure regulation have not been found to interplay earlier. According to our observation, increased levels of hepcidin could have a reducing effect on blood pressure via renin inhibition. It is also possible for the hepcidin-renin interaction to occur independently from the systemic hepcidin metabolism. Even though hepcidin expression occurs mainly in the liver, renal expression of hepcidin has been reported as well [32]. So far, elevated expression of renal hepcidin has been found to have an important role in acute kidney injury [30] but no possible role in the RAS system has suggested earlier.

Earlier, both low and high iron stores have been found to predict the poor prognosis of diabetic patients with coronary artery disease [33]. As low iron stores often lead to anaemia and thus diminished oxygen supply for cardiac muscle it seems logical to have a poor outcome with especially low ferritin levels. However, for high iron stores, the answer is not as straightforward. Several studies have reported high ferritin levels to have a relationship with hypertension [34–37]. The underlying mechanism has remained at least partially unclear although insulin resistance, fatty liver disease, and oxidative stress caused by excessive iron have been speculated to be mediators of the finding [35]. In the randomized controlled trial effect of phlebotomy on blood pressure was studied in patients with the metabolic syndrome. The group with phlebotomy-induced reduction of body iron stores had a 16.6 mmHg greater decrease of systolic blood pressure compared to the control group [38]. Therefore, it seems obvious to iron metabolism to be somehow involved in the origin of hypertension.

The effect of cardiovascular diseases on iron metabolism has been investigated recently. One of the most studied topics has been heart failure related iron deficiency with or without anaemia. Even 46% of the patients diagnosed with stable heart failure were found to have iron deficiency without anaemia according to IJsbrand et al [39]. In addition, iron depleted patients with heart failure seem to benefit from intravenous iron supplementation [40]. There are several and complex mechanisms behind heart failure related iron deficiency and at least renal failure, bone marrow resistance to erythropoietin, chronic inflammation, medication use, and hematinic deficiencies have been found so far [39].

Based on previous studies hepcidin seemed to be one of the possible factors linking iron metabolism to the blood pressure regulating system. We combined computational and laboratory methods to find out if hepcidin interacts with the RAS enzymes. Regarding the used methods there are some uncertainties involved in, but by combining several methods we wanted to minimise the artefact possibilities. The bio-layer interferometry technique was used to confirm the results from modelling and enzyme inhibition assays. According to literature, reported serum hepcidin concentrations differ around the world considerably, largely depending on the assay method used [7]. In Finland, serum active hepcidin levels vary in healthy controls from 0.4 to 16.8 nM (LC-MS/MS, www.hus.fi/en), and the lowest peptide concentration 10 nM used in our study represents a normal level of hepcidin in human circulation. However, for example in some inflammatory conditions the hepcidin levels may be significantly higher [41]. Therefore, we used 10 to 500 nM hepcidin in inhibition studies. However, we like to underline that the results of the study are primarily qualitative, and the quantitative examination is going to take place in the next phase.

In conclusion, we were able to show that hepcidin can bind to the active site of renin and inhibit its activity *in vitro*. Thus, hepcidin may be one factor involved in the regulation of blood pressure and this requires further research.
